# Lyophilization of Nanocapsules: Instability Sources, Formulation and Process Parameters

**DOI:** 10.3390/pharmaceutics13081112

**Published:** 2021-07-21

**Authors:** Ghania Degobert, Dunya Aydin

**Affiliations:** LAGEPP UMR 5007 CNRS, University of Lyon, Université Claude Bernard Lyon 1, 69622 Villeurbanne, France; dunya.aydin@etu.univ-lyon1.fr

**Keywords:** nanocapsules, freeze drying, unfreezable water, annealing, protectant, stability, hydrolysis, particle size, particle size distribution, storage

## Abstract

Polymeric nanocapsules have gained more and more interest in the medical sciences. Their core-shell structure offers numerous advantages, especially regarding their use as drug delivery systems. This review begins by presenting the different intrinsic sources of the instability of nanocapsules. The physical and chemical potential instabilities of nanocapsules reduce their shelf-life and constitute a barrier to their clinical use and to their commercialization. To overcome these issues, lyophilization is often used as a process of choice in the pharmaceutical industry especially when labile compounds are used. The state of the art of lyophilization nanocapsules is reviewed. The formulation properties and the process parameters are discussed for a complete understanding of their impact on the stability and storage of the final dried product. To assess the quality of the dried product, various characterization methods are also discussed.

## 1. Introduction

Over the last decades, nanoparticles have gained great attention in medical sciences [1,2,3] especially in the fields of imaging [4,5,6,7,8,9,10], sensing [11,12,13,14], gene delivery systems [15,16,17,18,19,20,21] and drug delivery [22,23,24,25,26,27,28,29,30]. Several studies have focused on the development of polymeric nanoparticles as drug delivery systems. Their ability to encapsulate various drugs, to deliver them in a sustained and/or targeted way and their ability to reduce the toxicity of some drugs by protecting non-targeted tissues make them extremely interesting [31,32]. Nanoparticles are submicron systems. Depending on the process used for their preparation nanoparticles, nanospheres or nanocapsules can be obtained [33,34,35]. Polymeric nanocapsules are spherical systems consisting of a core-shell whereas nanospheres consist of a regular sphere structure composed of a solid polymeric matrix [36].

Nanoparticles and more specifically nanocapsules (NCPs) are usually produced in aqueous suspension forms. The chemical and physical instabilities of such carriers in aqueous media are the major challenge. These instabilities constitute a barrier to their commercialization and do not ensure a long-term stability. To overcome the chemical and physical instabilities, the immobilization of the molecular mobility of the constituents of nanocapsule system is required. This immobilization can be achieved after drying [37,38,39,40,41].

Drying is one of the common unit operations in pharmaceutical industries permitting, through one or more phase transformations, to remove solvents (usually water) from liquid drug formulations in order to convert them into solid forms. The dry form allows to preserve their prime properties through the intended shelf-life. So, the obtention of solid-state nanocapsules is the main objective of lyophilization because it leads to the immobilization of the components of the formulation, namely the active substances, NCPs and the ligands if they are present at the surface of NCPs.

In this article, we will focus only on nanocapsules with an oily core. We will review the different instability sources of NCPs in aqueous medium and discuss why drying and more particularly lyophilization is the most convenient and applied process. Although lyophilization is the most suitable process, it can be nevertheless a source of stress at each stage of the lyophilization cycle. In order to overcome the stresses and to be successful at freeze drying nanocapsules, the impact of the formulation and the process is reviewed and discussed.

## 2. Instability of Nanocapsules in Aqueous Media

First of all, it is important to point out clearly the different intrinsic sources of the instability of polymeric NCPs. This instability can be either chemical or physical or both.

**Figure 1:**
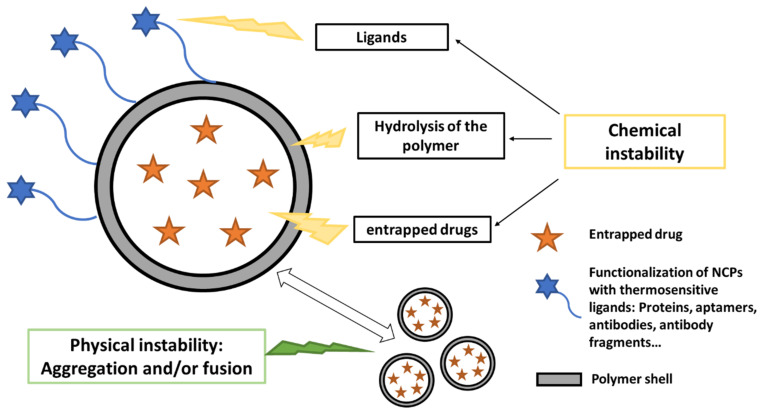
Sources of instability of nanocapsules requiring drying.

The physical and chemical instabilities cited in Figure 1 are not exhaustive but allow the reader to pay attention to all the components of NCPs that potentially may induce a certain instability. This can only be done through a knowledge of the physical and chemical properties of NCPs. This first step of preformulation constitutes the very essence of the work of the formulator.

The chemical instability of NCPs is mainly related: (i) to the hydrolysis of the polymer material forming the shell, (ii) to the instability of the entrapped drug, (iii) to the instability of thermosensitive ligands grafted at the surface of NCPs. The physical instability is often due to the aggregation and/or the fusion of NCPs. This constitutes a major obstacle to the storage in aqueous media. The drying process is therefore necessary to improve the stability of NCPs if only one of these instabilities is present.

### 2.1. Physical Instability

As the particle size of a suspension sample decreases down to the nanoscale, the surface to volume ratio and the surface energy increase. In the same way, NCPs tend to be inherently thermodynamically unstable and thus subjected to physical instability resulting in an aggregation and/or fusion of particles [42]. As a consequence, an increase in particle size and particle size distribution (PSD) is of paramount importance, particularly when NCPs are intended to the intravenous route. It is well known that aggregation and fusion of nanoparticles are frequently observed after a long period of storage [43,44,45,46]. The stability of poly(lactic-co-glycolic acid) (PLGA) nanoparticles suspensions was studied by Chacon et al. [44]. During a storage of 6 months at room temperature, the suspensions exhibited an aggregation process. In another study, indomethacin-loaded NCPs and nanospheres suspensions of PLA showed an aggregation after 3 months of storage at 50 °C. After 3 months of storage at room temperature, NCPs suspensions of PLA also exhibited aggregation [45]. The aggregation/fusion of the particles may fragilize the integrity of the particle, and lead to potential drug leakage. This phenomenon represents a risk to the health in the case of intravenous administration of a drug-loaded nanoparticle suspension, leading to blockage and embolism [46].

### 2.2. Chemical Instability

NCPs are usually formulated into aqueous media. The hydrolysis of the polymer material forming the shell is the most common source of chemical instability [47,48]. Indeed, the shell is usually made by hydrolysable polymers such as polyesters since they are FDA approved [49,50]. The hydrolytic action of water on the polymer constituting the shell leads to its degradation, thus potentially leading to drug leakage [51].

In Table 1 presents examples of polyester-based polymers used in the preparation of oily core NCPs. The low thickness of polymeric membrane (between 1.5 and 35 nm) is clearly shown. It is easily understood that due to its thinness, the polymeric membrane can be easily disrupted, impacting the stability and resulting in the loss of the spherical structure of NCPs and leakage of the encapsulated drug. Beyond the degradation of the polymer, the membrane constituting the shell is a fragile component of NCPs which may enhance the damage.

**Figure 2:**
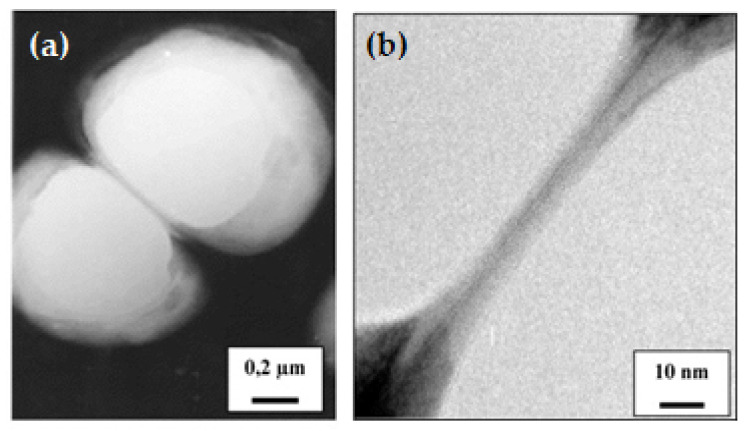
TEM observations of adjacent NCPs: (**a**) medium-sized magnification image; (**b**) high magnification image of the area of contact between the two NC. The membrane thickness of NC is estimated to be 1–2 nm. Adapted with permission from ref. [52]. 2021, Elsevier.

Figure 2 shows TEM observations of PCL NCPs membrane, being very narrow compared to the core. It is also noticeable on these images that NCPs can deform when they are in close contact, showing that the spherical shape is not rigid because of the liquid payload inside [52].

The chemical reactivity of the encapsulated drug and the thermosensitive ligands of the functionalized at NCPs surface can also contribute to the chemical instability of the NCPs. The encapsulation of potentially unstable drugs such as peptides, proteins, enzymes, or other active substances may be delicate [55,56,57,58]. Lastly, NCPs can be functionalized by various bioactive ligands able to interact with targeted biomolecules for achieving drug accumulation at tumor targeting regions. The presence of bioactive molecules on the surface of NCPs may also be a source of instability and leads to changes of the physical and the chemical properties of nanocapsules [59]. Furthermore, these molecules have specific properties, and thus may undergo destabilization or degradation. The previous sources of instabilities in NCPs are linked to their intrinsic properties. However, extrinsic parameters linked to environment such as light, water, pH, temperatures, etc., may also impact their stability. These parameters are not discussed in this review, but have been widely discussed in the literature [60,61,62].

It can be concluded that the physical and chemical instabilities of NCPs in aqueous media is a real challenge to be overcome. They are constantly prone to being destabilized, which limits their use. Another compromising limit is their reduced shelf-life. Several studies on the storage of nanosuspensions showed that their shelf life can be very low: suspensions of indomethacin-loaded NCPs are stable for 7 months, suspensions of diclofenac-loaded NCPs for 8 months, and suspensions of indomethacin-loaded nanospheres for 6 months [63]. However, for pharmaceutical products, a shelf-life of several years is required to commercialize a product. Regarding all the previous elements, it is essential to find ways to improve the NCPs stability and increase their shelf-life.

## 3. How to Improve the Stability and Storage of Nanocapsules?

To improve the stability and the storage conditions of nanoparticulate systems, freezing is widely used. Current examples which illustrates this preservation mode are mRNA-lipid nanoparticle COVID-19 vaccines of which the storage temperature is very low [64,65,66]. This particular case did not constitute an obstacle to its marketing given the global health context, but articles in the literature are currently looking to understand the mechanisms of instability of these nanoparticles to propose the most suitable drying process [60].

Therefore, converting NCPs suspensions into a solid form seems to be an obvious solution. For this purpose, it is therefore necessary to dry the product in order to remove water and to obtain dried nanocapsule product. This strategy would significantly improve the chemical and physical stability of NCPs. Different drying methods of NCPs have been reported in the literature such as spray drying [67,68,69], spray freeze drying [70,71,72,73], freeze drying [43,51,74,75]. Freeze drying and spray drying are the most conventional drying methods in pharmaceutical industry. Spray drying is widely used for drying particulate systems [45,68,74,75,76] and other products intended for aerosolization [77,78,79]. This process involves the atomization of a liquid into droplets drying process in hot environment. For products that are specifically heat-sensitive, freeze drying and spray freeze drying are the most convenient. However, spray freeze drying is a recently developed drying process that involves elements of spray drying and freeze drying. It is highly experimental and scaled for laboratory purpose [79]. Table 2 summarizes succinctly the advantages and the disadvantages of each drying process.

**Figure 3:**
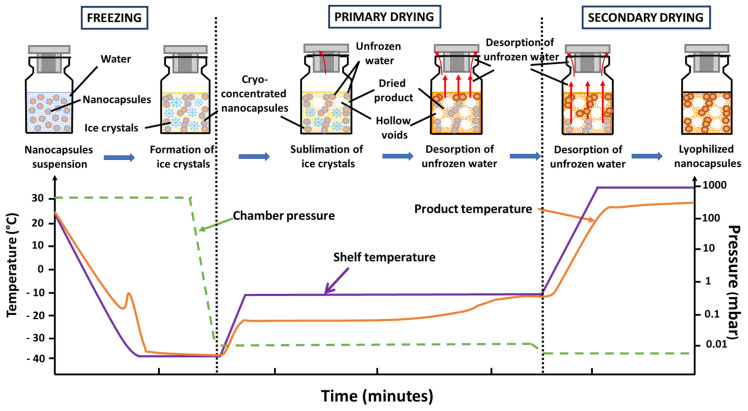
Successive stages in lyophilization: the evolution of the process parameters and the corresponding state of the product to be lyophilized.

Freeze drying, also known as lyophilization, is the most employed process for manufacturing pharmaceutical products that are thermo-sensitive and unstable in aqueous medium to ensure stability and prolonged storage in the dried state. Lyophilization process is a desiccation process where the solvent is frozen and then removed by sublimation under vacuum. Concretely, freeze drying can be divided into three stages: freezing (solidification), primary drying (ice sublimation) and secondary drying (desorption of unfrozen water). Figure 3 presents the evolution of the process parameters and the corresponding state of the product to be lyophilized during the successive stages in lyophilization.

To ensure an optimal execution of the drying process, it is necessary to determine the thermophysical properties of the product beforehand. Indeed, properties such as eutectic temperature (Teu), the glass transition temperature of maximally cryo-concentrated solute (T_g_’), the collapse temperature (T_c_), and glass transition temperature of the dried product (T_g_) are considered as critical. These thermophysical properties will guide the selection of the process parameters to develop the lyophilization cycle and to obtain a dried product with the highest quality (Table 3). The thermophysical properties of the product at each stage of the lyophilization process are defined in the next sections for a better understanding.

### 3.1. Freezing

Freezing is the first stage in the lyophilization process. During this step, the preparation is cooled, and ice nucleation of pure water occurs. As the freezing process continues, more and more water molecules contained in the preparation begin to grow into ice crystals. This leads to an increase of the concentration of solutes thus inducing an increase of the viscosity which reaches values of 10^11^ to 10^12^ Pa.s. This highly concentrated and viscous liquid solidifies and inhibits the further crystallization of ice. The preparation at this moment is segregated generally into a crystalline phase of ice crystals and an amorphous phase containing solutes. The temperature at which this vitreous transition appears is called glass transition temperature of maximally cryo-concentrated solute (T_g_’). For solutes that crystallize, the temperature of the system must be below the eutectic temperature to insure crystallization of the eutectic mixture (T_eu_) [80]. Supercooling is an undesirable phenomenon which can occur when freezing. At the end of this stage, water that remains in the solute at a liquid state and does not freeze at this temperature is called unfrozen water. The freezing stage occurs at atmospheric pressure.

### 3.2. Primary Drying

The primary drying stage involves sublimation of ice from the frozen product. In this process, (i) heat is transferred from the shelf to the frozen solution. (ii) The ice sublimes and the water vapor formed passes through the dried portion of the product (drying starts at the top of the vial to the bottom). At the end of the sublimation stage, a porous plug is formed. The pores correspond to the spaces that were occupied by ice crystals. During this stage, the pressure is reduced. Pikal et al. [25] recommend that the product temperature (T_p_) needs to be controlled with a safe margin (2–5 °C) below the collapse temperature (T_c_) or the glass transition temperature (T_g_’) in the case of very unstable biomolecules. The collapse temperature is determined by the microscopic observation (see Section 5.1.6) during primary drying using freeze drying microscopy (FDM) [39].

### 3.3. Secondary Drying

Secondary drying involves the removal of adsorbed water from the product. It concerns the unfrozen water which did not separate out as ice during the freezing stage. The molecular motion of water in the solid and its evaporation to achieve lower residual water may require a greater energy to tear them from the solid by imposing a higher temperature and a lower pressure than during the sublimation stage. To maintain the product in its glassy state, the product temperature must be kept below T_g_.

In conclusion of this part, for each stage, the process parameters necessary to develop a lyophilization cycle, the set temperatures applied must be as close as possible to the temperature of product to be freeze dried (T_p_).

## 4. Freeze Drying Nanocapsules

Lyophilization is a suitable process in improving stability of NCPs. In general, a good lyophilizate should maintain the physical and chemical properties of the prime product: a cake with good aspect, short reconstitution time, low residual moisture and a long-term stability. NCPs must be easily resuspended and must not present significant modifications of particle size, particle distribution size (PSD) and preserve the activity of the drug encapsulated [31]. To achieve these goals, optimization of the formulation, optimization of the process and the storage conditions of the lyophilized product are essential. Table 4 shows the encapsulated drug and the conditions of lyophilization NCPs prepared using different types of polymers. In the following sections, we will focus our attention on the formulation and the lyophilization process.

### 4.1. Formulation

Although lyophilization is the most suitable process, unfortunately, it may induce numerous stresses that could destabilize the nanocapsules. The stresses can occur at different stages of lyophilization and may affect the formulation and thus the stability of dried NCPs. For these reasons, the formulation has to be deeply studied before the lyophilization process. The freezing and primary drying stages can be aggressive for NCPs. During the freezing stage of the nanocapsule suspension, a phase separation occurs between the formation of ice and the cryo-concentration of the suspension in the interstis. The high concentration of NCPs may generate their aggregation or fusion [39]. During the drying stage, the removal of ice and unfrozen water may also destabilize the particles [31].

#### 4.1.1. Protectant

To prevent the instability of NCPs induced at the different stages, two types of protectants can be used: cryoprotectants and lyoprotectants. Cryoprotectants are defined as excipients whose primary function is to protect the active constituent and NCPs during the freezing process, whereas the lyoprotectants stabilize them during both freezing and drying stages. In the lyophilization process, most of protectants used are sugars such as mannitol [86], trehalose [84], sucrose [43], glucose [44] or lactose [86] as presented in Table 5. Sugars are preferable, mainly because they are chemically innocuous, and can be vitrified during the freezing stage for most of them. Sugars are effective cryoprotectants only when used at relatively high concentrations [37]. Usually, cryoprotectants may also act as lyoprotectants [31]. Polymers are also used as protectant [39,85,88].

PCL NCPs showed aggregation and the formation of macroscopic particles after lyophilization without cryoprotectant [39]. For ovalbumin-loaded chitosan NCPs, lyophilization without any cryoprotectant induced the formation of macroscopic aggregates [86].

The immobilization of NCPs within a glassy cryo-concentrated matrix of cryoprotectant lowers the mobility of molecules and thus can prevent their aggregation and protect them against the mechanical stress of ice crystals. In general, the freezing step should be carried out below all the thermal events of the frozen sample to ensure its total solidification. Abdelwahed et al. [39] demonstrated the importance of the vitrification of cryoprotectants on the stabilization of NCPs during freezing. Differential scanning calorimetry (DSC) thermograms of NCPs suspensions prepared with five different types of cryoprotectant were realized. Cryoprotectants used in this study were sucrose, glucose, hydroxypropyl β-cyclodextrin (HPβCD), polyvinyl pyrrolidone, and mannitol. The analysis revealed that NCPs suspension has different glass transition temperatures depending on the type of cryoprotectant used in the formulation. This shows that NCPs can be included within vitrified glasses of cryoprotectants during the freezing stage. Abdelwahed et al. [39] also pointed out that lyophilization with mannitol as a cryoprotectant induced aggregation of the NCPs. Mannitol crystallizes forming eutectics with ice, thus leading to a phase separation in the cryo-concentrated portion of the frozen suspension with no opportunity for a stabilization interaction with NCPs. Hence, annealing is usually applied to the formulations in order to monitor the crystallization of mannitol and avoid vial breakage [91,92]. Another way to inhibit crystallization of mannitol is also the addition of co-solutes. In fact, a mannitol solution at 10% (*w/v*) with and without 1% NaCl was conducted and showed that the presence of NaCl inhibits the crystallization of mannitol, which remains in amorphous state during freezing with NaCl. Beyond the protectants role to protect the product over the process, they may also have an impact on the drying stages. Indeed, their presence in the formulation must be kept in mind because they may modify the collapse temperature (T_c_) of the product. Such phenomenon can impact the product quality, increase the time of reconstitution and increase also the residual moisture content. PCL NCPs have been freeze dried with four different excipients: sucrose, glucose, PVP, and HPβCD. The determination of collapse temperature (T_c_) by FDM revealed that these different formulations have different collapse temperatures [39]. The collapse temperature of formulations prepared with sucrose, glucose, HPβCD and PVP corresponds to −30.86, −42.70, −15.43 and −22.06 °C, respectively. Furthermore, the modification of the collapse temperature of the formulation influences the time of sublimation. The higher the collapse temperature, the faster the sublimation rate is.

Depending on their type and on their concentration, protectants do not have the same level of stabilization. Different parameters of freeze-dried ovalbumin loaded chitosan NCPs were studied before and after lyophilization: [86] diameter measurement, polydispersity index, ratio of NCPs size, aspect, reconstitution time and residual moisture content. Several cryoprotectants were tested: glucose, lactose, mannitol, sucrose, and mannitol with polymers, varying their concentration in order to assess their impact on the characterization of NCPs. The values corresponding to the previous parameters clearly demonstrate how the type and the concentration of cryoprotectants have an impact on the final freeze-dried product. Only a few lyophilizates showed correct appearances. All of them include mannitol, regardless of the concentration or the addition of concomitant polymer. Additionally, the residual water contents show different values depending on type and concentration of cryoprotectant. NCPs suspensions freeze dried with mannitol 10% (*w/v*) showed a residual moisture content of 1.30%, whereas the collapsed formulation without cryoprotectant had a residual moisture content of 4.10%. According to the authors, NCPs with mannitol 10% (*w/v*) demonstrated a successful lyophilization regarding the characterization parameters and the preservation of porous structures of the freeze-dried cake observed by scanning electron microscopy.

The stabilization of NCPs not only depends on the concentration of the cryoprotectant, but also on the concentration of NCPs. Chitosan NCPs recovery of the initial properties upon lyophilization and reconstitution varied with the concentration of NCPs (0.25, 0.5 and 1% *w/v*) and cryoprotectant (5 and 10% *w/v*). The best results were obtained with a NCPs concentration of 0.25% *w/v* and trehalose concentrations of 10% *w/v*, most likely due to the more adequate protection of the NCPs by the vitrified matrix of the cryoprotectant against the stress of the freezing stage [84]. It is therefore essential to choose the adequate cryoprotectant at the right concentration. Date et al. [93] demonstrated the use of freeze–thaw as a simple and quick approach to select the most appropriate cryoprotectant. Freeze–thaw study is based on the principle that an excipient, which protects nanoparticle during the first stage of freezing, is likely to be an effective cryoprotectant. Nanoparticles of rifampicin were prepared by emulsion-solvent diffusion method and were then frozen with different concentrations of fructose and trehalose as cryoprotectants. The study revealed that the cryoprotection increased with the concentration of cryoprotectant and that freezing temperature did not influence the freeze–thaw study. The freeze-dried nanoparticles showed good redispersibility with an increase of their size with 20% *w/v* of trehalose and fructose.

As for the lyoprotective effect, the mechanism of action is based on the formation of hydrogen bonds of the protectant with the polar groups at the surface of nanoparticles at the end of the drying process. Protectants preserve the native structures of nanoparticles by serving as water substitutes [51]. The amorphous state of NCPs and a lyoprotectant allows maximal hydrogen bonding between NCPs and stabilizer agent molecules. The crystallization of this stabilizer can limit the formation of hydrogen bonds [39]. Protectants not only protect NCPs during the process of lyophilization, but they can also have an impact on their stability during storage. The temperature storage of freeze-dried NCPs should be kept at a temperature below the glass transition temperature of the formulation in order to maintain the glassy state of the protectant and prevent aggregation the nanocapsules [39].

#### 4.1.2. Nanocapsule Stabilizers

To ensure the maximum stability, a stabilizing agent is often used in NCP formulations since they are colloidal systems. It should be localized at the nanoparticles surface. The use of a stabilizer in the formulation of NCPs suspension can improve their stability and prevent their aggregation. In general, stabilizers are used at a concentration between 2.5 and 10%. During storage, aggregation becomes an even more important stability issue. For these reasons, the selection of an appropriate stabilizer and at the right concentration are crucial parameters of stability [94]. Various polymers for stabilization of NCPs are numerous. Polyvinyl alcohol (PVA) is one of the most common stabilizers used to produce nanoparticles, allowing the production of stable nanoparticles with small size and narrow distribution [31,43,95]. Despite repeated washing of the nanocapsules, a fraction of PVA remains associated at the particles surface and may improve their freezing resistance [51]. Its remaining presence may thus influence the lyophilization process. Abdelwahed et al. [43] reported that PCL NCPs formulated with 2.5% (*w/v*) and 5% (*w/v*) of PVA were stable with no significant aggregation upon lyophilization, with no cryo- or lyoprotectant added.

Nevertheless, the presence of surfactants during the lyophilization process does not always lead to a stabilization effect. Chitosan NCPs of docetaxel were freeze dried and reconstituted. Upon reconstitution, the NCPs showed an increase in the size value of 50–100 nm. However, those containing poloxamer showed a significant increase in their size, a result that suggested that poloxamer interferes in the lyophilization process probably by its crystallization during freezing [84].

#### 4.1.3. Core and Encapsulated Drug

Freeze–thawing stability of PCL NCPs are prepared with 1.25% of PVA protectant and by using two oils with different melting temperatures (Miglyol: Tm −3.5 °C and silicone oil: Tm −100 °C). The freezing temperature of −50 °C applied solidified the miglyol and kept the silicone oil in a liquid state. The preservation of NCPs containing silicone oil in the liquid state was not improved by the freezing. The encapsulated drug must also be taken into consideration. During the lyophilization process, the drug release kinetics may be affected, as well as drug degradation or drug leakage may occur. NCPs prepared with PLA as polymer and benzyl benzoate as oil were freeze dried [96]. However, after the re-hydration of freeze-dried NCPs, a loss of drug as large as 50% was observed, probably due to changes in the structure of the capsule wall or because water crystallization stress could break the NCPs, promoting the leakage of their contents into the continuous phase. In another study [81], lyophilized Olmesartan Medoxomil (OM) oily-core NCPs (ONC) filled in hard gelatin capsules (HGC) showed the highest percentage of drug dissolution and release. After one and a half hours, the lyophilized OM-ONC released 99.67 ± 0.97% while only 40.85 ± 5.07% and 55.83 ± 12.2% was released from pure OM powder HGC and marketed tablet product, respectively. Compared to the OM-ONC colloidal dispersion, which has not been lyophilized, the dissolution profile of lyophilized NCPs is almost identical. The drug release study also showed that the lyophilization process did not adversely affect the enhanced dissolution profile of OM-ONC colloidal dispersion. Furthermore, the dissolution of OM is slightly improved. This may be attributed to the reduction in particle size after lyophilization and evaporation of residual oil from the formulation.

### 4.2. Freeze Drying Process

After optimization of the formulation, it is necessary to use adequate lyophilization parameters in order to obtain a lyophilizate with high quality. The lyophilization process is divided is three main stages: freezing (solidification), primary drying (ice sublimation) and secondary drying (desorption of unfrozen water). Nevertheless, this process induces many stresses and can damage the product. It is therefore essential to understand the mechanisms inducing these instabilities at the different stages of the process. This permits to make an informed choice of the formulation and the process parameters. The critical formulation properties include the glass transition temperature of the frozen sample (T_g_’), the collapse temperature of the formulation (T_c_), the stability of the capsule and its encapsulated drug, and also the properties of the excipients used [51].

Abdelwahed et al. showed the importance of knowing thermal properties of the formulation prior to lyophilization. Lyophilization of PCL NCPs using mannitol as a cryoprotectant induced the aggregation of the particles [39]. The same results for mannitol were observed for the thermal analysis of chitosan NCPs: from DSC heating curve, the crystallization of mannitol and unfrozen water could be observed [86]. These results confirm that it is essential to know the thermal properties of the components of the formulation in order to ensure a correct lyophilization. Sucrose, glucose and trehalose have been used as protectant for the lyophilization of PCL itraconazole-loaded nanospheres [96].

In another study, PCL nanoparticles were characterized during the freezing and lyophilization stages without cryoprotectant. It was observed that the lyophilization process was faster with slow cooling rates rather than with high cooling rates. At slow cooling rates, a small number of large ice crystals are obtained, whereas at high cooling rates, a large number of small ice crystals are obtained. Increasing the porosity and permeability of the dry matrix can lead to an increased sublimation rate, shortening the drying time [97,98]. For the highest cooling rates, nanoparticles became more and more aggregated, showing that the cooling rate is extremely important in preventing NCPs from aggregation during lyophilization. Additionally, results of mean particle size between freeze–thawing and lyophilization were not significantly different. This demonstrates that the freezing stage can break the nanoparticles and lead to the leakage of their content [99]. For chitosan NCPs with mannitol 10% (*w/v*), four cooling procedures were applied: immersion in liquid nitrogen, at −196 °C, immersion in acetone/dry ice bath at −78 °C, and two ramps temperatures of 1 or 2.5 °C/min. The results of size particle, polydispersity index and ratio of NCPs size after and before lyophilization being near 1 show that these nanocapsules were very stable during all the freezing procedures [86].

However, the presence of a cryoprotectant can impact the nanoparticles during freezing. Depending on the type and the concentration of the cryoprectant used in the formulation, the stabilization of NCPs over lyophilization can be modified. During lyophilization of griseofulvin-lipid nanoparticles with xylose as cryoprotectant, the crystallization of the saccharide induced an irreversible aggregation [100]. Lee et al. [101] demonstrated that a fast cooling rate and high cryoprotectant concentration generates better redispersibility of dried nanoparticles. However, under certain conditions, this statement is not always verified. In the case of sucrose, the addition of 10% to the preparation actually promotes the aggregation of nanoparticles at a high cooling rate. On the contrary for Polyethylene glycol (PEG) above 15%, when the concentration was increased, the nanosuspensions could be freeze dried into redispersable powders regardless of the cooling rate. This study confirms that the selection of cryoprotectants, with the freezing conditions, are crucial in the stabilization of NCPs during lyophilization because they have an impact on nanoparticles aggregation and redispersibility. For purified mannan-coated cationic nanoparticles, a fast freezing without any cryoprotectant led to less aggregation than slow freezing. However, the rate of freezing did not impact the particle size of the thawed nanoparticles when a cryoprotectant was added to the preparation [102]. In another study [90], the addition of cryoprotectants, namely trehalose, sucrose and glucose, enabled to maintain the properties of PLGA and PCL nanoparticles after freeze–thawing, regardless of the cooling conditions and polymer type.

Freezing, being the first step in the lyophilization process, strongly influences the size and the shape of ice crystals, thus impacting primary and secondary drying. Usually, the optimization of the lyophilization process focuses on decreasing the time of the primary drying because it is the longest stage.

**Figure 4:**
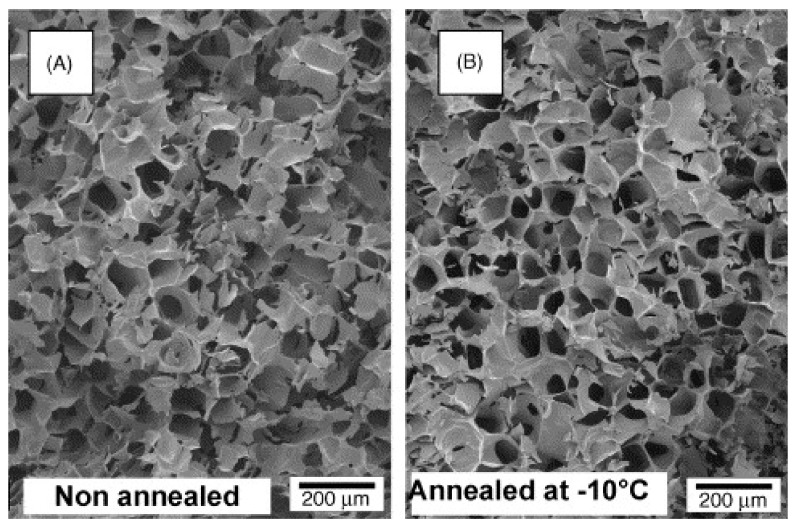
Structure of freeze-dried PVP without annealing (**A**) and with annealing at −10 °C (**B**). Note that the two plugs had a leafy amorphous appearance consisting of an irregular array of large pores which had been previously occupied by ice (magnification 60 ×). Adapted with permission from ref. [104]. 2021, Springer Nature.

Annealing is a process stage in which samples are maintained at a specific subfreezing temperature, above T_g_’ for a period of time [103]. Annealing can be useful for many reasons. First, it can help in reducing heterogeneities in drying rates. It is crucial that all vials have similar processing conditions. However, several factors such as heterogeneous nucleation, vial condition, and positional variations in the lyophilizer may generate ice nucleation of the samples within the vials at different temperatures. This results in potential heterogeneities in drying rates and therefore product quality. Annealing can thus eliminate variations in initial ice crystal size distributions induced by variable nucleation temperatures [103]. Annealing can also be used to increase the primary drying rate during the lyophilization cycle, and therefore shorten the entire cycle time. Indeed, annealing causes an increase in the size of ice crystals, leading to an increased diameter of pores previously occupied by these crystals and an accelerated primary drying [31]. Figure 4 shows scanning electron microscopy images of freeze-dried PCL NCPs without annealing (on the left) and with annealing at −10 °C (on the right). The annealed samples present larger pores, that were previously occupied by ice.

**Figure 5:**
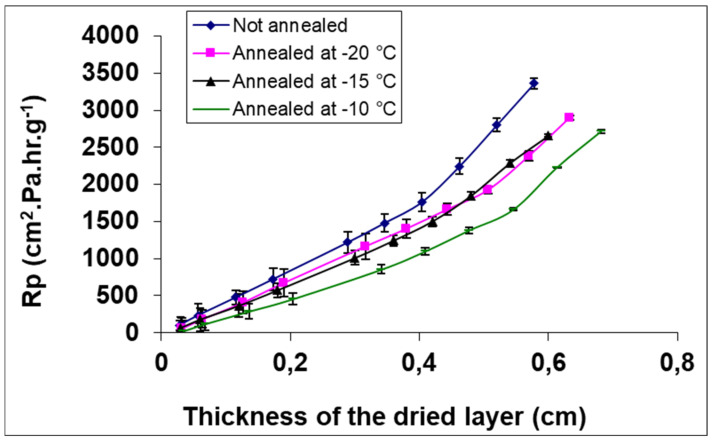
Resistance to mass transfer as a function of freeze-dried layer thickness for formulated PVP at different annealing temperatures. Adapted with permission from ref. [104]. 2021, Springer Nature.

In addition, annealing can inhibit the formation of the skin layer on the top of the freeze-dried product, which increases the sublimation rate. Indeed, this skin is formed by the migration of cryoprotectants and nanoparticles during the crystallization of ice. This layer may slow down the transfer of water vapor during sublimation [51]. Figure 5 shows the water vapor transfer resistance (Rp) as a function of dried layer thickness of PVP freeze-dried solution without annealing and with annealing at three different temperatures.

One can observe that the resistance increases with the increase of the dry layer thickness. Additionally, the resistance to water vapor transfer reduces significantly when annealing is applied. The lower resistance was observed with annealing at −10 °C. This result may be attributed to the fact that annealing generates an increase in pore size, therefore reducing the resistance to water vapor transfer during sublimation [105]. Furthermore, morphological changes induced by annealing may also impact reconstitution. Searles et al. [103] observed the formation of holes in the skin layer on the surface of annealed freeze-dried products and this may allow a better liquid penetration.

Abdelwahed et al. [104] studied the impact of annealing on the drying stage during lyophilization of PCL NCPs. To assess the influence of annealing on sublimation rate, NCPs prepared with two cryoprotectants (sucrose and PVP) were freeze dried by applying four different freezing protocols: freezing without annealing and freezing with annealing during 1 h at three different temperatures: −10, −15 and −20 °C.

The results demonstrated that the process which did not include annealing had the lowest sublimation rate for both protectants. On the contrary, annealed samples had the highest sublimation rates. Additionally, sublimation rates increased when the temperature of annealing was increased from −20 to −10 °C. Nevertheless, the increase of sublimation rates was not the same for both protectants because they have different glass transition temperatures. Indeed, the temperatures chosen for annealing were above the T_g_’ of sucrose than of PVP. They also studied the impact of annealing on the secondary drying and showed that is it dependent on the type of cryoprotectant used: in the case of PVP, the annealing step did not slow down the secondary drying kinetic, whereas with sucrose, a longer kinetic was observed.

The influence of temperatures of annealing on particle sizes of chitosan ovalbumin-loaded nanocapsules prepared with 10% (*w/v*) of mannitol was studied [86]. The T_g_’ of the formulation is about −34 °C. Four different annealing temperatures from −5 to −25 °C during 1 h have been applied. An increase in the nanocapsules size ratio before and after annealing (SF/SI) was observed. For annealing temperatures of −25, −15, −10 and −5 °C, the corresponding size ratios were 1.15, 1, 1.68, 1.92, and 2.31, respectively. This demonstrates that an increase of annealing temperature leads to an increase of nanocapsules size. However, the freeze-dried preparations did not present any sign of collapse or shrinkage. It is important to point out that the morphological aspect of the freeze-dried product is not necessarily linked to a successful lyophilization.

At the end of primary drying, adsorbed water from the product remains. This water did not separate out as ice during the freezing stage, and hence, did not sublimate during primary drying. It can be adsorbed on the surface of the crystalline product, or is in the solute phase either as hydrate water in a crystalline hydrate or dissolved in an amorphous solid to form a solid solution [106]. This water is removed during the secondary drying step.

Generally, the secondary drying is carried out at a higher shelf temperature than primary drying. High temperatures are necessary to reduce the residual moisture content to optimal values. This process is called isothermal desorption: the bound water is desorbed from the product [107]. However, this stage must be carefully conducted to gradually increase the temperature, but also to avoid any damage to the lyophilized product, such as shrinkage or collapse. A fast temperature ramp might cause collapse of amorphous products. At the beginning of the secondary drying stage, the residual moisture content is high, and the glass transition temperature is low. Therefore, the potential for collapse is quite high [97].

This second drying stage is crucial to reduce the moisture content of the product to an acceptable level. The final residual moisture of the freeze-dried product requires to be precisely controlled. The objective of secondary drying is to reduce the residual moisture content to a level optimal for stability, which is usually less than 1%. Indeed, high water content normally decreases the storage stability of drugs [97,98].

### 4.3. Storage Stability

Several studies have shown that lyophilization could be an interesting strategy to improve the nanocapsules stability. Ovalbumin loaded nanocapsules prepared from 6-O-carboxymethyl chitosan have been successfully freeze dried [86], as well as docetaxel loaded chitosan nanocapsules [84], PCL and Eudragit nanocapsules [108]. Freeze-dried polymeric nanocapsules could be very stable after six months of storage at accelerated conditions according to ICH guidelines (1993), showing no signs of collapse or shrinkage, and also preserving their properties over the stability study when using convenient protectant [39]. Yacasi et al. [109] showed that flurbiprofen-loaded PCL nanocapsules have a good stability for 365 days over storage stability studies at room temperature, with no significant changes in particle size, polydispersity index, encapsulation efficiency, zeta potential and residual moisture content values. Chitosan NCPs containing docetaxel, a drug used for cancer treatments, have shown that NCPs maintained the antiproliferative effect of the drug and have not been affected by the lyophilization process [84].

Beyond improving the stability of NCPs, lyophilization also aims to establish their shelf-life to ensure their efficacy and safety. For this purpose, certain factors must be taken into consideration. The glass transition temperature, especially of the polymer constituting the shell, represents an important parameter during storage. Table 6. shows the Tg of a few polymers. Indeed, it determines the physical state of the polymer and consequently will affect the stability of the suspension during storage [110]. However, referring to Figure 1, the polymer constituting the shell is not the only important factor in the stability of NCPs. The stability of the encapsulated substance, as well as the functionalized groups if they are present will also play a major role during the storage of freeze-dried NCPs.

Nevertheless, the glass transition temperature of the anhydrous polymer is not necessarily the same as the glass transition temperature of the resulting freeze-dried polymeric nanoparticle. For example, a lower T_g_ for freeze-dried PLGA nanoparticles was observed compared to pure PLGA [113].

Another important parameter during storage is the residual moisture content in the lyophilized product. In the formulation, water may affect the formulation by acting as a plasticizer. This can lead to a significant decrease of the glass transition temperature of the polymer or other excipients in the formulation [114]. For instance, the Tg of PLGA nanoparticles in suspension is lower (32.1 °C) than the Tg of the same nanoparticles in freeze dried form (35.5 °C) [113]. Indeed, water may interact with polar polymer chain groups like carboxyl groups existing in the polymer’s structure or interfere with intermolecular polymer chain bonds. This results in an increased polymer chain mobility and a decreased Tg. Therefore, in lyophilized NCPs, the residual moisture content is a critical parameter since it may affect the glass transition temperature of the formulation and can consequently affect the stability of the formulation over storage. A small amount of water up to 3.7 mg/g was able to decrease the Tg (37.3 °C) of freeze-dried PLGA nanoparticles in comparison to the Tg of the completely dried nanoparticles (47.2 °C) [113]. A slight change of the glass transition temperature of the polymer near the storage temperature may affect its physical state, and thus its degradation [110].

Additionally, it has been demonstrated that a high residual moisture content can destabilize nanoparticles upon storage by inducing the crystallization of the cryoprotectant. After 6 months of high relative humidity of 75 ± 5%, freeze-dried PCL NCPs showed the crystallization of their cryoprotectants, namely sucrose and glucose. Indeed, the residual moisture content may shift the Tg of the formulation to below the temperature of storage, therefore causing the crystallization of the formulation [39].

The humidity content of a freeze-dried formulation may significantly vary during storage. To predict the water activities in the product, it is important to get the knowledge of the moisture sorption isotherms of the lyophilized formulations [115]. To prevent product collapse upon storage, it is essential to maintain the lyophilized product in a glassy state, and therefore to store them below their glass transition temperature. Formulations with high glass transition temperature are preferable [114].

## 5. Characterization Methods

In this section, various characterization methods are discussed. These characterization techniques are essential whether they are used before or after lyophilization. Before lyophilization, some parameters are important to determine in order to perform a correct process. After lyophilization, other characterization methods are necessary to assess the quality of the final dried product.

### 5.1. Morphological Observations

#### 5.1.1. Macroscopic Aspect

**Figure 6:**
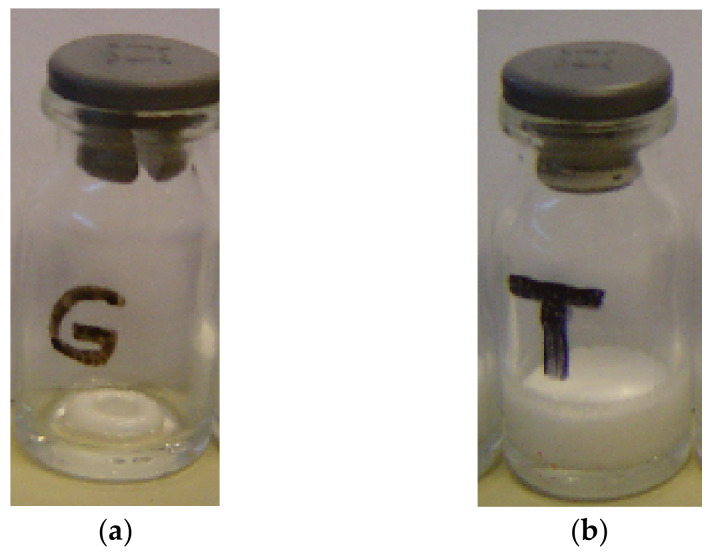
Photographs of collapsed and non-collapsed freeze-dried (**a**) sucrose (T_c_ = −30 °C) and (**b**) PVP (T_c_ = −22 °C).

One of the first analysis of a freeze-dried product is its macroscopic aspect. Visually inspecting the lyophilized product allows to assess events such as collapse, shrinkage, change of texture or color, etc. These may indicate potential changes in the properties of NCPs. The final freeze-dried product should occupy the same volume as the original frozen mass [51]. Figure 6 shows photographs of freeze-dried collapsed sucrose (a) and non-collapsed freeze-dried PVP (b).

One can observe the collapsed and shrunken aspect in Figure 6a compared to Figure 6b. This demonstrates that a simple macroscopic observation of the lyophilized cakes can give information if the lyophilization process has been carried out correctly.

#### 5.1.2. Scanning Electron Microscopy (SEM)

The microscopic observation of a lyophilized product allows to visualize the microstructure of the freeze-dried matrix and to assess the preservation of the nanoparticles’ integrity and morphology. For lyophilized nanoparticles, certain electronic microscopic techniques can be used.

**Figure 7:**
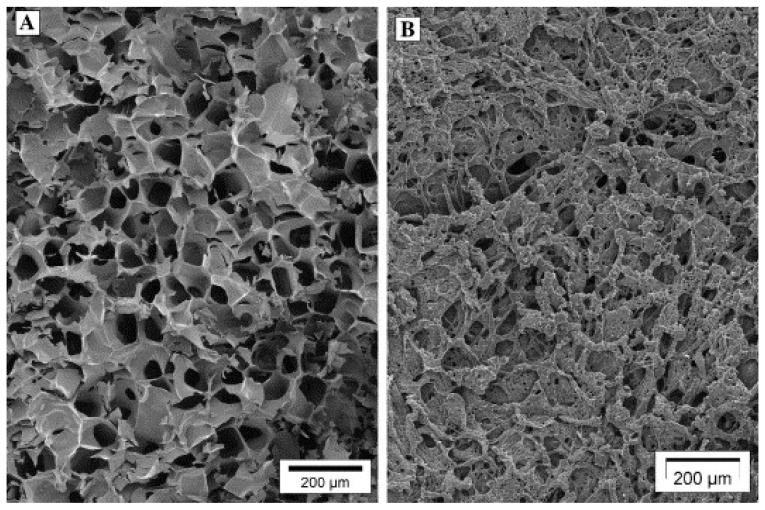
Scanning electron micrographs of freeze-dried (**A**) PVP and (**B**) sucrose. Note the partial collapse of sucrose and the apparition of holes in the structure whereas the plates of PVP are intact. Scale bar: 200 μm. Adapted with permission from ref. [39]. 2021, Taylor & Francis.

Analysis by SEM at low magnification allows to observe the porosity of the lyophilized product and signs of collapse. Figure 7 shows scanning electron micrographs of freeze-dried PVP and sucrose preparations. One can observe the dried PVP is structured into intact plates with the conservation of porous structure (Figure 7A). On the other hand, dried sucrose develops holes in the structure, which indicates a small collapse (Figure 7B) [39].

**Figure 8:**
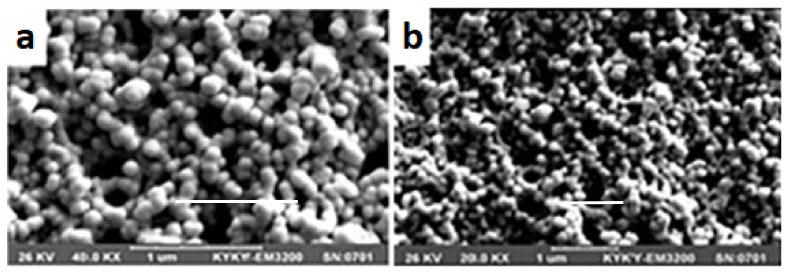
SEM micrographs of chitosan coated calcium-alginate nanocapsules fresh sample immediately after preparation (**a**); resuspended dried sample (**b**). Adapted with permission from ref. [55]. 2021, Taylor & Francis.

SEM enables the analysis of the surface topography, roughness and morphology of solid particles at high resolution [116]. Figure 8 shows SEM micrographs of chitosan coated calcium-alginate NCPs loaded with liraglutide in different forms. In suspension after preparation (Figure 8a) and resuspended freeze-dried sample (Figure 8b), it is possible to distinguish the nanocapsules [55].

However, SEM does not allow the observation of freeze-dried NCP samples. Usually, a cryoprotectant is added to the formulation to protect NCPs upon lyophilization. Therefore, at a certain percentage of protectant, NCPs are entrapped in its matrix and are not visible.

#### 5.1.3. Environmental Scanning Electron Microscopy

**Figure 9:**
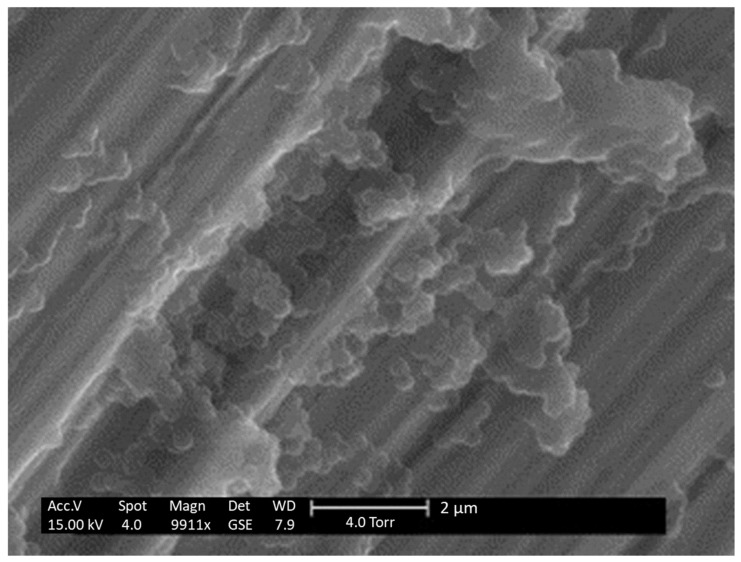
ESEM imaging of freeze-dried purified PCL NC after reconstitution prepared with 5% (*w/v*) of HPβCD as cryoprotectant. Adapted with permission from ref. [51]. 2021, Elsevier.

Environmental SEM (ESEM) on the other hand uses a lower vacuum and allows the analysis of hydrated specimens without any sample preparation. Therefore, ESEM enables the observation of lyophilized NCPs in a hydrated state. Figure 9 shows ESEM images of freeze-dried PCL NCPs with HPβCD as cryoprotectant. The NCPs are spherical and monodisperse, demonstrating that they are correctly conserved after lyophilization [39].

#### 5.1.4. Transmission Electron Microscopy (TEM)

Transmission electron microscopy (TEM) allows to obtain high magnification images via an electron beam directed through the sample. This technique gives information about particle shape, and more specifically enables the observation of the membrane constituting the polymeric shell and measurement of its thickness. Figure 2 shows TEM micrographs of PCL NCPs and their membrane. The authors could estimate the membrane thickness to 1–2 nm for one nanocapsule. As for cryogenic TEM, this technique enables the analysis of the sample in frozen state.

#### 5.1.5. Atomic Force Microscopy (AFM)

Another interesting, but non-electronic microscopic observation is atomic force microscopy (AFM). This technique generates images by scanning a cantilever over the surface of a sample. It provides nanoscopic three-dimensional images of the surface topography, heterogeneities and local mechanical behavior [116]. This technique allows to determine particle size, shape and surface structure.

#### 5.1.6. Freeze Drying Microscopy

During freeze drying microscopy (FDM), the sample corresponding to a droplet around 20 µL is first frozen under atmospheric pressure, followed by an increase of temperature and pressure. This technique enables the assessment of the collapse temperature of the dried structure. The collapse temperature is determined by visually recognizing morphological changes at the sublimation front of the sample [117].

### 5.2. Reconstitution Time

To reconstitute the lyophilized product, the same volume of water that had been removed should be added. Usually, a correct lyophilizate should be rehydrated immediately. However, in the case of collapsed formulations, a long reconstitution time can be observed. To achieve the re-suspension of lyophilized NCPs after the addition of water, manual shaking is usually used [51].

### 5.3. Particle Size and Polydispersity Index

The analysis of particle size after reconstitution is important to assess the stability of NCPs after lyophilization. The preservation of NCPs diameter after lyophilization is considered as a good indication of a successful process. Furthermore, the ratio of nanoparticles size before and after lyophilization can be calculated. A value close to one indicates the conservation of the size. On the contrary, a high ratio value shows an aggregation of particles [51]. The particle size can be measured by dynamic light scattering (DLS), also known as photon correlation spectroscopy. Particle sizes of PCL NCPs were measured by photon correlation microscopy using different cryoprotectants. The formulations using sucrose, HPβCD, glucose and PVP showed a ratio of NCPs size before and after lyophilization near one, confirming the conservation of the NCPs size after rehydration. On the other hand, NCPs freeze dried with mannitol produced macroscopic particles that were non-measurable, and formulations with mannitol + 1% NaCl exhibited a ratio of 3.5, indicating a significant increase in particle size after lyophilization [39].

The polydispersity index gives information about the distribution of NCPs size. The values before and after lyophilization can be compared in order to evaluate the conservation of nanoparticles distribution. The PDI is a dimensionless calculated number based on the mean particle diameter [118].

### 5.4. Zeta Potential

The measurement of the zeta potential is useful to evaluate the state of NCPs surface and to detect any eventual modification after lyophilization. The zeta potential value is closely related to suspension stability and particle surface morphology [119]. Its value can be determined by light scattering techniques such as electrophoresis light scattering (ELS), also known as Doppler microelectrophoresis [118]. The zeta potential can also be used to assess the interactions between nanoparticles and excipients. De Chasteigner et al. [96] found that the addition of 10% of sucrose to itraconazole loaded PCL nanospheres suspensions decreased the surface charge from −40.9 to −20.4 mV. In fact, the nanosphere surface is masked because of hydrogen bonds between OH groups of the cryoprotectant the nanosphere surface. After lyophilization, the surface charge is more decreased, due to a rearrangement of the surfactant at the nanoparticles surface.

### 5.5. Activity of Encapsulated Substances and/or Functionalized Ligands

The measurement of the drug content before and after lyophilization is important to assess any leakage of the drug during the process. It can be measured by high performance liquid chromatography (HPLC) [51]. After lyophilization, it is important to assess that the freeze-dried NCPs conserved their activities. The activity of both the encapsulated active substance and/or the functionalized molecules at the surface if they are present must be evaluated. Depending on the type of active substance encapsulated and/or functionalized molecules present at the surface, various techniques can be used to evaluate their activities.

Freeze-dried docetaxel-loaded chitosan NCPs were developed to evaluate their potential for intracellular delivery of docetaxel and their efficacy [84]. For this purpose, cell viability assays were realized after incubation of lyophilized and reconstituted NCPs with the cells. The results showed that these NCPs were able to enter the cells and deliver the encapsulated drug intracellularly. Furthermore, they were able to maintain the IC50 values (measurement of the effectiveness of the substance) previously observed for the free drug and non-freeze-dried NCPs. These results demonstrate that the NCPs were not altered by the lyophilization process, and that they are effective for the intracellular delivery of docetaxel.

In another study [81], lyophilized Olmesartan Medoxomil-loaded PCL NCPs were formulated to study the enhancement in the oral bioavailability of the drug. To evaluate the drug’s activity, an in vivo comparative pharmacokinetic study in rats was conducted. The maximum plasma concentration of the drug after oral administration of reconstituted NCPs was 37.66 µg/mL, compared to 19.56 µg/mL for the oral marketed tablet. Additionally, this maximum plasma concentration for NCPs was recorded at a shorter time (2 h) than marketed tablets (4 h). These results indicate a significant improvement for the in vivo behavior of the drug in reconstituted lyophilized NCPs. When heat-sensitive products are encapsulated or grafted onto the surface of the NCPS, the stabilization hypothesis cited in the literature is that of the replacement of hydrogen bonds of water molecules by the hydrogen bonds of sugars [41,120,121].

### 5.6. Residual Moisture Content and Study of Water Sorption Isotherms

The stability of NCPs is strongly related to the residual moisture content of lyophilized product. This parameter is important to determine because it may influence the thermal and the structure properties of the lyophilized product. The residual moisture content can be determined by Karl Fischer titration or thermogravimetric analysis [122]. However, Karl Fischer titration method is usually preferable. Indeed, the thermogravimetric analysis is based on weight loss of the product during heating. This requires the opening of vials and the exposure of the lyophilized product to the ambient atmosphere. This may result in potential sorption of water by the product and incorrect resulting values of residual moisture content. Karl Fischer titration method allows to work with sealed vials and therefore gives more accurate values. NIR spectroscopy can also be used to analyze residual moisture in lyophilized pharmaceuticals sealed in glass vials [123].

Additionally, the study of sorption isotherm of water is essential to determine the degree of hygroscopicity of the product, and also to assess the ease in the secondary drying [51]. To conduct a water sorption study, the product is stored in desiccators with solid salt or saturated salt solutions until the establishment of equilibrium at an ambient temperature. The salts may be phosphorus pentoxide, lithium chloride, potassium acetate, magnesium chloride, potassium carbonate, and sodium chloride, which generate relative humidity of approximately 0%, 11%, 23%, 33%, 43%, and 75%, respectively. Dynamic vapor sorption (DVS) technique can also be used to measure the sorption and/or desorption of water vapor on a powder [116].

### 5.7. Differential Scanning Calorimetry

As previously mentioned, the NCPs suspension should be frozen below the T_g_’, the drying stages and storage should also occur below the T_g_’. For this purpose, evaluating the T_g_’ of the formulation by thermal analysis is essential. It can be performed by differential scanning calorimetry (DSC). Besides, this technique can be useful to evaluate the interactions between protectants and NCPs [51]. DSC also enables to detect interactions between drug and polymer components, and changes in drug crystallinity after the formulation process. Thermograms by DSC of olmesartan medoxomil (drug), PCL (polymer), mannitol (cryoprotectant) and lyophilized Olmesartan-Mexodomil loaded PCL NCPs prepared with mannitol were realized. The drug, the polymer and the cryoprotectant all exhibited endothermic peaks corresponding to their melting points. However, the thermogram of the freeze-dried formulation showed only two peaks for PCL and mannitol. The absence of the characteristic peak of the drug is attributed to the transformation of its crystalline form to amorphous form, and its solubilization inside the oily core of the nanocapsule [81].

## 6. Conclusions

The use of nanocapsules in drug delivery is well established. This review showed that the chemical and physical instabilities of nanocapsules constitute a major barrier to their clinical use and to their commercialization. The use of lyophilization becomes necessary especially when NCPs contain hydrolysable or thermolabile substances (polymer, encapsulated drug and/or functionalized ligand at the surface of NCPs). Lyophilization is a multidisciplinary field. It requires deep knowledge of formulation, physical and chemical characterizations of the product and finally a deep knowledge of the whole process. In the literature, it has been shown that the formulation and the lyophilization process are intimately related and one impacts the other. In fact, lyophilization of nanocapsules is quite delicate because the process generates numerous stresses on these fragile systems. In order to withstand these stresses and perform an optimal lyophilization cycle, a convenient protectant at the right concentration can be used. The annealing can also be applied to shorten the duration of sublimation stage. The lyophilization of nanocapsules, when it is fully controlled could really revolutionize their use in the medical field by ensuring stability and a long-term preservation. Currently, the research is in progress in order to set up a continuous lyophilization to reduce the cost [122,123].

## Figures and Tables

**Table 1:** Examples of nanocapsule diameters with oily core and membrane thicknesses.

Composition Polymer Shell/Oily Core	Nanocapsule Diameter (nm)	Membrane Thickness (nm)	Reference
PCL/Miglyol^®^ 812	520	1.5–2	[52]
PCL65/BB	228 ± 16.1	20.9	[53]
PCL100/BB	241.7 ± 32.5	22.2	
PLGA/BB	228.8 ± 9.8	20.9	
PLA/BB	236.6 ± 13.2	21.8	
PCL80/Miglyol^®^ 812	457 ± 5	35	[54]

PCL65, PCL80 and PCL100: poly-ε-caprolactone mol. wt. 65,000; 80,000 and 100,000 g/mol respectively; PLA: poly(d,l-lactide) mol. wt. 200,000; PLGA: poly(lactic-co-glycolic acid) mol. wt. 40,000; BB: Benzyl benzoate.

**Table 2:** Advantages and limitations of the following processes: freeze drying, spray drying and spray freeze drying.

Drying Procedure	Advantages	Disadvantages
Freeze drying	- Low temperature of the process adapted to heat-sensitive product - Closing vials under inert gas adapted to oxygen-sensitive product - Accurately dosed - High porosity induces a short reconstitution time - Controlled moisture content - Aseptic process	- Long processing time - Expensive set up - Complex process - Maintenance cost - Exposure to ice–water interface
Spray drying	- Rapid drying process - Convenient system - Low cost - Particle engineering - Good flowability of powders - Aerosolizable powder	- Yield (50–70%) - Unsuitable for oxygen-sensitive product - Unsuitable for heat-sensitive product
Spray freeze drying	- Rapid freezing - Particle engineering - High yield - Good flowability of powders - Aerosolizable powder	- Long processing time - High cost - Fragile particles - Complex process - Requires liquid nitrogen

**Table 3:** Presentation of the process parameters and the product thermophysical properties at each stage of freeze drying and required characterization techniques for the final dried product.

		Process Parameters		Formulation before FD	Characterization of Lyophilized NCPs
Stages		Temperature	Pressure	Thermal Characterization	
Freezing		Cooling temp.	Atmospheric pressure	T_P_ < T_eu_, T_g_’	Particle size and size distribution Moisture content and sorption isotherms Reconstitution time Zeta potential and polydispersity index Drug content Thermal analysis Microscopic electronic observations (SEM, TEM) Activity of active substances and functionalized ligands
		Cooling rate			
		Duration *			
Annealing *		Heating rate		T_P_ > T_g_’	
		Annealing temperature			
		Duration *			
		Cooling rate			
Primary drying (sublimation)		Duration *	Reduced pressure	T_P_ < 2–5 °C of T_c_ or T_g_’	
		Heating rate			
		Sublimation temp.			
Secondary drying (desorption)		Desoption temp.	Reduced pressure	T_P_ < T_g_ ^#^	
		Heating rate			
		Duration *			

* Duration: The duration is important to ensure the completion of each stage for all the vials. ^#^ Considering the drug encapsulated (proteins, enzyme, etc.) and the functionalized ligands (cf. Figure 1).

**Table 4:** Examples of freeze-dried nanocapsules and process conditions.

		Encapsulated Drug	Conditions of Lyophilization Process			References
Polymer			Freezing	Primary Drying	Secondary Drying	
PCL		Olmesartan Medoxomil	−40 °C overnight	Different drying phases for about 48h *		[81]
PCL		Vitamin E	−45 °C for 120 min	−20 °C for 480 min	+20 °C for 360 min	[82]
Chitosan + dextran sulphate		IutA protein from *Escherichia coli*	−80 °C	−40 to −20 °C for 35 h	Gradually increase temperatures up to +20 °C	[83]
Chitosan		Docetaxel	Quickly frozen in liquid nitrogen	−35 °C for 60 h	24 h in high vacuum	[84]
Hyaluronic acid		Docetaxel	−20 °C	−35 °C for 60 h 50 mTorr	0 °C for 24 h	[85]
6-O-carboxymethyl chitosan		Ovalbumin	−50 °C for 30 min	−40 °C for 6 h	20 °C for 4 h	[86]
Isobutylcyanoacrylate		Iodized oil	Liquid nitrogen	−90 °C for 48 h under 10 mPa *		[57]
PCL		Fish oil	−30, −20 and −10 °C	−50 °C under 0.05 mbar *		[87]

* No further information on the drying stages.

**Table 5:** Examples of freeze-dried nanocapsules and protectants most widely used in literature.

Polymer	Encapsulated Substance	Protectants	References
6-O-carboxymethyl chitosan	Ovalbumin	Mannitol, lactose	[86]
Chitosan	Docetaxel	Trehalose	[84]
PCL	no drug	Sucrose	[43]
PLGA	Cyclosporine	Glucose	[44]
PCL	no drug	PVP	[39]
PLGA	Ciprofloxacin HCl	Dextran	[89]
PLGA	Insulin	Fructose	[88]
PCL	Cyclosporin	Sorbitol	[90]

**Table 6:** Glass transition temperatures (T_g_) of anhydrous polymer materials.

Polymers	Glass Transition Temperatures	References
PLA	60–65 °C	[111]
Chitosan	140–150 °C	[112]
PCL	−60 °C	[111]

## Data Availability

Not applicable.
